# A biopsychosocial perspective on endometriosis: the importance of psychological inflexibility

**DOI:** 10.1007/s00404-025-08276-0

**Published:** 2026-02-24

**Authors:** Sophia Åkerblom, Ingrid Peppler Jönsson, Åsa Ringqvist, Johanna Nordengren, Xiang Zhao

**Affiliations:** 1 Department of Pain Rehabilitation, Skåne University Hospital, Lund, Sweden; 2 Department of Health Sciences, Lund University, Lund, Sweden; 3 Department of Clinical Sciences, Lund University, Malmö, Sweden; 4 Department of Obstetrics and Gynecology, Skåne University Hospital, Malmö, Sweden; 5 Institute of Psychology, University of Klagenfurt, Klagenfurt Am Wörthersee, Austria; 6 Research Institute of Humanities and Social Sciences, University of Sharjah, Sharjah, UAE

**Keywords:** Endometriosis, Biopsychosocial model, Psychological inflexibility, Pain catastrophizing, Kinesiophobia

## Abstract

**Introduction:**

Treatment strategies for endometriosis have traditionally been biomedical. There is a need for a more multidimensional understanding of endometriosis and more targeted and individualized treatment interventions, including psychological approaches.

**Methods:**

The aims of this study were twofold: (1) to identify key biopsychosocial characteristics in individuals attending a tertiary clinic for endometriosis and (2) to inform the development of future, targeted, and efficacious interventions by examining the importance of psychological processes central to two scientific models, pain catastrophizing and fear of movement from the fear-avoidance model, and psychological inflexibility from the psychological flexibility model.

**Results:**

Psychosocial variables, more specifically perceived control and powerlessness, social support, and depression, were of particular importance to the symptom structure in this patient population. In contrast, biological factors appeared to have low relevance within this network. When aiming to inform the development of future, promising psychological interventions for endometriosis, psychological inflexibility emerged as the most important psychological process variable in the symptom network.

**Conclusions:**

A multidimensional approach based on the biopsychosocial model appears valuable for understanding endometriosis. Treatment interventions grounded in the psychological flexibility model may hold promise for this patient population, a possibility that warrants further investigation in future studies.

## What does this study add to the clinical work?

**Table:** 

A multidimensional approach based on the biopsychosocial model seems to be useful in understanding, assessing, and treating endometriosis. Psychological treatments and in particular interventions based on the psychological flexibility model, such as Acceptance and Commitment Therapy, may be effective for individuals living with endometriosis	

## Introduction

Endometriosis is an inflammatory disease in which endometrial-like tissue grows outside the uterus, affecting an estimated 10% of women worldwide [1, 2]. Characteristic symptoms include painful menstruation, ovulation, intercourse, and defecation; urinary tract and gastrointestinal symptoms; and fertility problems [1]. A significant proportion of individuals with endometriosis experience chronic pelvic pain, and pain is widely recognized as a cardinal symptom of the condition [1, 3, 4]. Endometriosis can also lead to far-reaching social and psychological consequences, including psychiatric comorbidities, such as depression and anxiety, reduced quality of life, and negative effects on key life areas, such as relationships, sexuality, fertility, work, and leisure [5–9]. The symptoms of endometriosis are complex, heterogeneous, and multidimensional, pointing to the fact that the symptom profiles might be better understood from a biopsychosocial viewpoint, where the dynamic interplay of biological, psychological, and social factors is considered together [10, 11].

Still, treatment strategies for endometriosis are typically biomedical, and a baseline treatment is often hormone therapy to reduce endometriosis-associated dyspareunia, dysmenorrhea, and non-menstrual pain, which cannot be tolerated by some due to side effects [12, 13]. Different analgesics for pain management are frequently prescribed, and in certain instances surgical methods are implemented. Surgical interventions can be of benefit to selected individuals and offer pain relief for some individuals [14, 15]. However, medical interventions are connected to a heightened risk of iatrogenic consequences and physical, psychological, and social adverse events, such as serious side effects, repeated surgery due to recurrence of pain, and worsened symptoms and quality of life [15–21]. Individuals with insignificant effects from these treatments are frequently prescribed long-term opioids for pain control, which has an associated risk of amplified pain from opioid-induced hyperalgesia, disability, tolerance, and heightened risk of addiction and overdose [22, 23].

Taken together, there is a need for more targeted and individualized treatment interventions for individuals with endometriosis, incorporating psychological, biological, and social factors [10, 24]. Calls have also been made for effective psychological treatment of endometriosis [24, 25], but thus far psychological research and treatment methods within the field have been relatively sparse, and further exploration is needed [12, 13, 26, 27].

Evidence-based treatment programs for chronic pain are grounded in the biopsychosocial model and commonly use psychological principles, most often cognitive behavioral therapy (CBT) [25]. CBT has proven to be effective for chronic pain and might be a promising candidate for improved quality of life in endometriosis [24, 25]. The fear-avoidance model is a focused CBT model centered on chronic pain [28]. Kinesiophobia or fear of movement, and catastrophizing are core concepts within this long-established and active scientific model [29–32]. The psychological flexibility model, which also stems from the CBT tradition, has gained increasing empirical support in the chronic pain field over the past decade. This growing support is largely due to accumulating evidence that greater psychological flexibility, characterized by openness to experience, present-moment awareness, and value-based action, is associated with improved adjustment and reduced disability among individuals with chronic pain [28, 33]. Kinesiophobia, pain catastrophizing, and psychological (in) flexibility have been established as key treatment mechanisms in interventions for chronic pain and have gained some attention in endometriosis [26, 28, 31, 34–37]. However, the psychological processes most relevant to endometriosis and the interventions likely to benefit affected individuals remain largely unknown [26, 27].

Complex relations among psychological, social, and biological factors can be captured using network analysis [38]. This statistical tool has been widely used within psychiatry and clinical psychology to outline symptom structures, identify important features of different conditions, and gain insight into important psychological mechanisms underlying specific conditions [39–42]. The aims of this study are twofold: (1) to identify key biopsychosocial characteristics in individuals attending a tertiary clinic for endometriosis and (2) to inform the development of future, targeted, and efficacious interventions by examining the importance of psychological processes central to two scientific models, pain catastrophizing and fear of movement from the fear-avoidance model, and psychological inflexibility from the psychological flexibility model.

## Methods

### Participants

Participants were 113 individuals with painful, either laparoscopically or ultrasound-verified endometriosis, referred for assessment at the endometriosis clinic at Skåne University Hospital between April 2019 and May 2024. The endometriosis clinic is part of the Swedish National Health Service, serving as a regional, specialist center providing healthcare services to individuals with endometriosis. The study was approved by the Swedish Ethical Review Authority (2019-00023) and participants provided informed consent.

### Measures

For analytical clarity, factors were grouped into biological, psychological, and social categories. This categorization provides a clear framework for interpreting the structure and patterns of associations in the network analysis, thereby facilitating a more meaningful understanding of how different domains of the biopsychosocial model interact in the context of endometriosis. However, some constructs (such as pain interference) encompass elements of more than one domain, consistent with the integrative nature of the model. In this context, psychological process factors, such as psychological inflexibility, represent underlying mechanisms that shape psychological adaptation and act as key targets for intervention within the scientific models examined.

#### Biological factors

Pain duration was registered in years.

Pain intensity was measured with *the Numerical Rating Scale (NRS).* The NRS is well-established and is used in pain research to measure pain intensity over the past week on an 11-point scale (0 = no pain; 10 = worst possible pain). The NRS detects variations in pain level in different contexts [43, 44].

Pain extent, or the number of pain locations, was assessed using 36 predefined anatomical areas (18 on the right side of the body and 18 on the left) and patients indicated the areas where they experienced pain: (1) head/face, (2) neck, (3) shoulder, (4) upper arm, (5) elbow, (6) forearm, (7) hand, (8) anterior aspect of chest, (9) lateral aspect of chest, (10) abdomen, (11) sexual organs, (12) upper back, (13) lower back, (14) hip/gluteal area, (15) thigh, (16) knee, (17) shank, and (18) foot. The number of pain locations (range: 0–36) was summed.

#### Social factors

Pain interference was measured using this specific subscale from *the Multidimensional Pain Inventory Version 2 (MPI)* [45]. Pain interference is rated on a seven-point scale (0 = never; 6 = very often) in the areas of family and marital functioning, work and work-related activities, and social and leisure activities. A mean interference score is computed, with higher scores denoting greater functional impairment from pain. The MPI has adequate psychometric properties [46] and the Swedish version used in this study has shown good sensitivity in pain outcome studies [47].

Social support and work life were measured with these specific subscales from *the Endometriosis Health Profile-30 (EHP-30).* The EHP-30 assesses health-related quality of life in individuals with endometriosis. Social support was measured using a main subscale of the EHP-30, whereas work life was assessed with a supplementary module subscale, which also covers additional areas, including relationships with children, sexual relationships, feelings about the medical profession, feelings about treatment, and feelings about infertility. The items are rated on a five-point scale (0 = never; 4 = always). For each domain, the scores are converted to a 0–100 scale, with 0 representing the best health-related quality of life and 100 the worst [48]. The psychometric properties of both the original and Swedish versions of the EHP-30 have been deemed to be valid and reliable [48, 49].

#### Psychological factors

Control and powerlessness and self-image were also measured with these specific subscales from *the Endometriosis Health Profile-30 (EHP-30)* as described above. Again, for each domain, the scores are converted to a 0–100 scale, with 0 representing the best health-related quality of life and 100 the worst [48].

Rates of anxiety and depression were assessed with the 14-item *Hospital Anxiety and Depression Scale (HADS)* [50], with seven anxiety and seven depression items, respectively. The items are rated from 0 to 3, with higher scores indicating greater levels of depression and anxiety over the past week. Scores of 0–7, 8–10, and 11–21 represent cutoff points for non-cases, doubtful cases, and clinical cases [50]. The psychometric properties of the English original and of the Swedish version used in this study are well-established and widely accepted [50, 51].

#### Psychological process factors

Catastrophic thinking was assessed using the 13-item *Pain Catastrophizing Scale (PCS)*, which evaluates three dimensions: helplessness, magnification, and rumination [52]. The items are scored on a five-point scale (0 = not at all; 4 = all the time) and are added to produce a total score, where higher scores represent greater catastrophizing. The psychometric properties of the measure are well-established [52].

Psychological inflexibility was assessed using *the Psychological Inflexibility in Pain Scale (PIPS)* [53]. The 12 items are scored on a seven-point scale (1 = never true; 7 = always true), where higher scores indicate a greater degree of psychological inflexibility. The psychometric properties of the Swedish PIPS are acceptable [53].

Fear of movement or reinjury (kinesiophobia) was measured using *the Tampa Scale of Kinesiophobia (TSK)* [54]. The 17 items are rated on a four-point scale (1 = strongly disagree; 4 = strongly agree). Higher scores indicate greater levels of kinesiophobia. Both the original and Swedish versions of the TSK have displayed satisfactory psychometric properties [54–56].

### Analysis

The study constructs were organized broadly following the biopsychosocial framework [10]. Biological factors included pain duration, pain intensity, and pain extent. Psychological factors were anxiety, depression, control and powerlessness, and self-image. Psychological process factors, pain catastrophizing and fear of movement (the fear-avoidance model) and psychological inflexibility (the psychological flexibility model) were also included here. Social factors included pain interference, social support, and work life. Given the cross-sectional nature of the data, undirected (rather than directed) networks were used to encapsulate the complex and reciprocal interplays among the biopsychosocial symptoms. Each symptom was regarded as a node in the system [57]. To estimate the connections (i.e., edges) between nodes, the EBICglasso algorithm was applied, which is based on an LASSO method [58]. In the visualization, thicker edges reflect stronger partial correlations; green and red links mean positive and negative associations, respectively. To relax the normality assumption in estimation, a nonparanormal transformation (“npn”) was also applied to normalize the data [59]. While the packages produce several centrality metrics for networks, we only used strength centrality, as it is considered the most interpretable metric in psychosomatic data [60]. The strength metric refers to a measure of centrality that considers the weights of the edges connected to a node: ${s}_{i}=\sum_{j\ne i}\left|{\omega }_{ij}\right|$, where ${\omega }_{ij}$ is the weight of the edge between nodes *i* and *j*. Because the absolute values of the weights are used, the metric reflects the overall magnitude of connections, regardless of their positive or negative direction. Thus, the nodes with relatively larger strength values possess more direct connections and stronger partial correlations with other nodes. In our context, nodes with larger strength values represent the most influential symptoms in the biopsychosocial networks. To examine the stability of networks and centrality values, we used 1000 bootstraps for each edge as well as for the strength metrics. Based on visual inspection, most visible and strong edges did not include zero in the bootstrapped networks. Even when the subset sample comprised 40% of the original sample, the strength estimate remained above 0.70. The packages “qgraph” [61] and “bootnet” [62] were used to estimate and visualize the networks using R. Pairwise deletion was used to handle missing data.

## Results

### Descriptive information of sample

The study included 113 women. On average, participants were 36.9 years (SD = 8.0), with ages ranging from 21 to 61 years. The majority of participants (70.8%) were born in Sweden or other Nordic countries. Regarding education, 62.5% of participants had completed tertiary education, while 27.7% had completed secondary education. The average reported duration of pain was 12.1 years. Participants reported an average pain extent of 9.7 locations. For more details, see Table 1. Using the supplementary parts of the EHP-30 participants rated the following areas if relevant to them: work life, relationship with children, sexual relationship, feelings about the medical profession, feelings about treatment, and feelings about infertility (Table 2). Taken together, work life, sexual relationship, and feelings about treatment appeared to be the subscales most relevant to the individuals in this study. A minority felt that the children and infertility subscales were relevant to them. The subscales reflecting the worst health status were sexual relationship, feelings about treatment, and feelings about infertility.

**Table 1:** Means, standard deviations and minimum and maximum values for the sample on the network variables

Variable	Mean	SD	Min	Max
INT	5.63	2.74	0	10
EXT	9.71	6.71	0	36
DUR	12.10	10.17	0	34
INF	3.62	1.49	0	6
DEP	7.87	4.42	0	19
ANX	10.29	4.67	0	21
WOK	45.00	28.40	0	100
SUP	50.35	27.30	0	100
CON	63.01	25.97	0	100
IMG	56.00	27.78	0	100
PIF	57.83	14.54	19	84
CAT	31.86	12.28	1	52
FOM	39.65	9.99	19	62

*INT* pain intensity (NRS), *EXT* number of pain locations, *DUR* pain duration, *INF* pain interference (MPI), *DEP* depression (HADS), *ANX* anxiety (HADS), *WOK* work life (EHP-30), *SUP* social support (EHP-30), *CON* control and powerlessness (EHP-30), *IMG* self-image (EHP-30), *PIF* psychological inflexibility (PIPS), *CAT* pain catastrophizing (PCS), *FOM* fear of movement (TAMPA)

**Table 2:** Descriptive information from the EHP-30 supplementary modules

Subscore	Valid cases (%)	Mean	SD
Work life	76 (67.3%)	45.00	28.40
Relationship with children	49 (43.4%)	34.69	25.29
Sexual relationship	73 (64.6%)	59.45	31.42
Feelings about the medical profession	66 (58.4%)	40.15	27.10
Feelings about treatment	70 (61.9%)	57.02	25.13
Feelings about infertility	41 (36.3%)	55.79	30.89

### Networks and centrality metrics

**Fig. 1:**
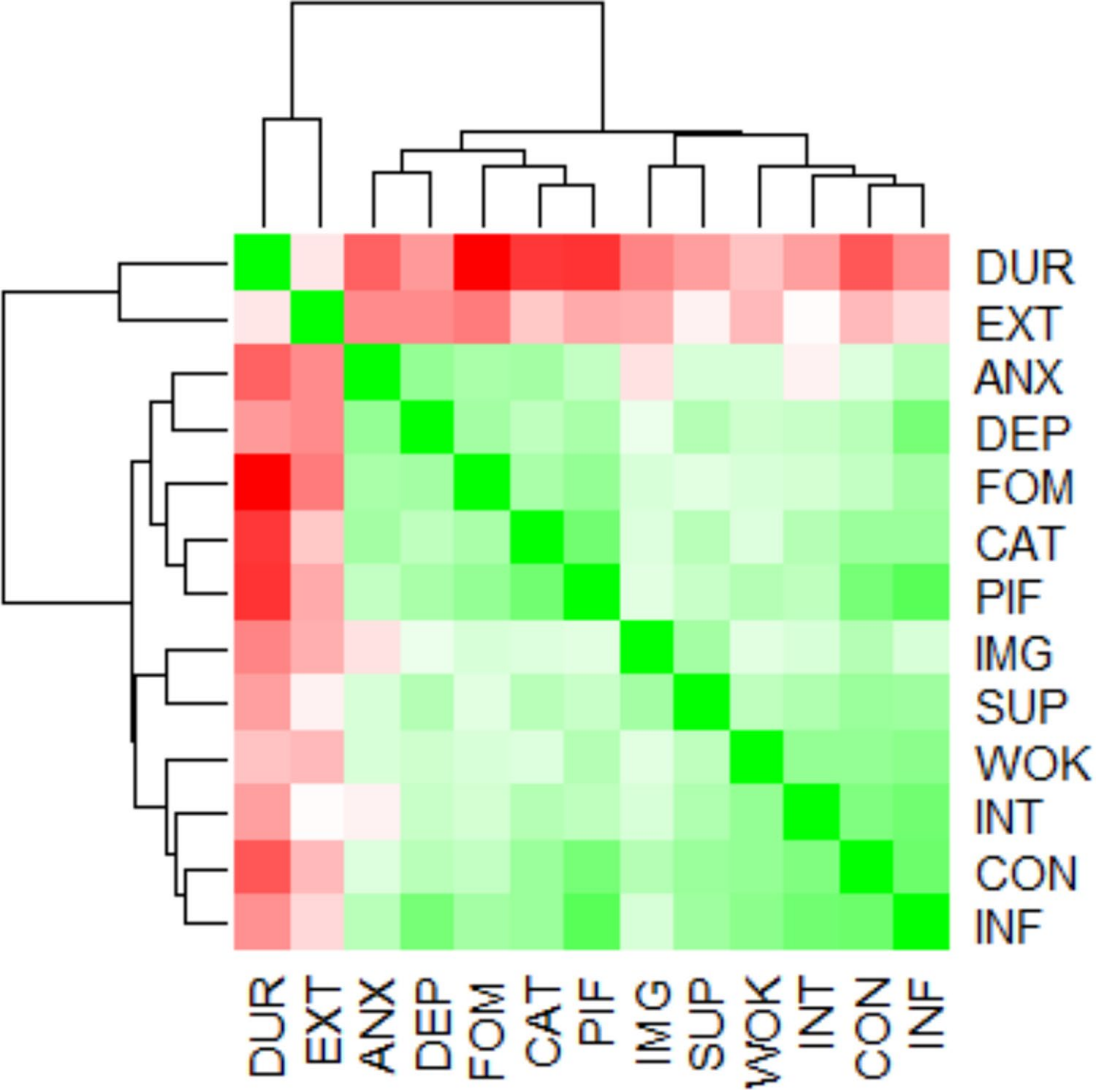
Heatmap with a dendrogram. *INT* pain intensity (NRS), *EXT* number of pain locations, *DUR* pain duration, *INF* pain interference (MPI), *DEP* depression (HADS), *ANX* anxiety (HADS), *WOK* work life (EHP-30), *SUP* social support (EHP-30), *CON* control and powerlessness (EHP-30), *IMG* self-image (EHP-30), *PIF* psychological inflexibility (PIPS), *CAT* pain catastrophizing (PCS), *FOM* fear of movement (TAMPA)

Bivariate Spearman’s correlations were first examined and visualized using a heatmap (Fig. 1). Apart from the intercorrelations among the EHP-30 subscales, the remaining variables only showed sporadic associations with other variables. Notably, the dendrogram on the sides of the heatmap briefly delineates the clustering of nodes. Three main clusters emerged: (1) a *biological cluster*, encompassing pain duration and pain extent; (2) an *emotions and psychological processes cluster*, which includes depression, anxiety, catastrophizing, psychological inflexibility, and fear of movement; and (3) a *pain-related cluster*, consisting of pain interference and intensity, along with four EHP-30 subscales: work life, social support, control and powerlessness, and self-image.

**Fig. 2:**
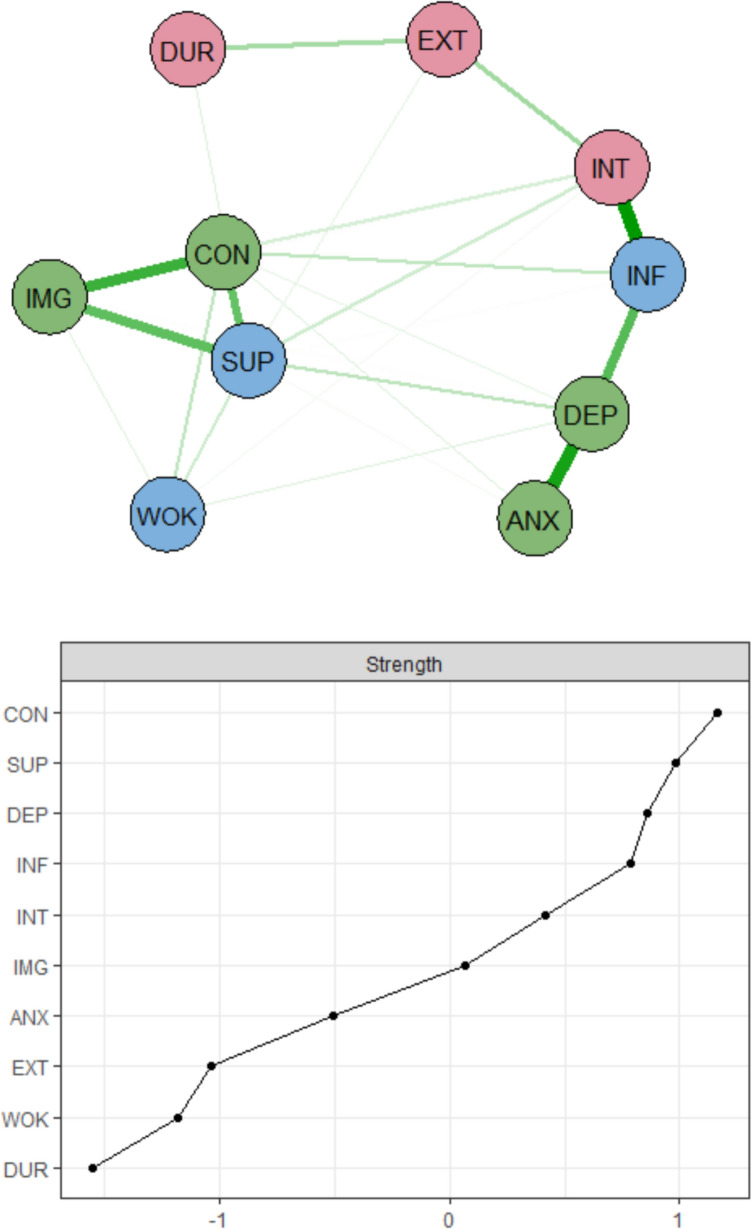
Network with strength centrality plot. There are three domains in nodes: red = biological nodes, green = psychological nodes, blue = social nodes. In the strength centrality plot, *z*-scores are shown on the *x*-axis rather than raw centrality indices. *INT* pain intensity (NRS), *EXT* number of pain locations, *DUR* pain duration, *INF* pain interference (MPI), *DEP* depression (HADS), *ANX* anxiety (HADS), *WOK* work life (EHP-30), *SUP* social support (EHP-30), *CON* control and powerlessness (EHP-30), *IMG* self-image (EHP-30)

As illustrated in the first biopsychosocial network (without the psychological process factors), pain interference, pain intensity, anxiety, and depression were closely connected, forming a cluster comprising pain aspects and negative emotions. Three subscales (control and powerlessness, social support, and self-image) of the EHP-30 formed another close cluster (Fig. 2), the EHP-30-based cluster. In terms of centrality, biological factors showed limited relevance within the network, with pain extent and pain duration displaying low strength values. In contrast, control and powerlessness and social support demonstrated multiple, strong connections with other nodes. As shown in the centrality plot, control and powerlessness had the largest strength values in the network, followed by social support and depression. All of these variables were classified as psychological or social nodes.

**Fig. 3:**
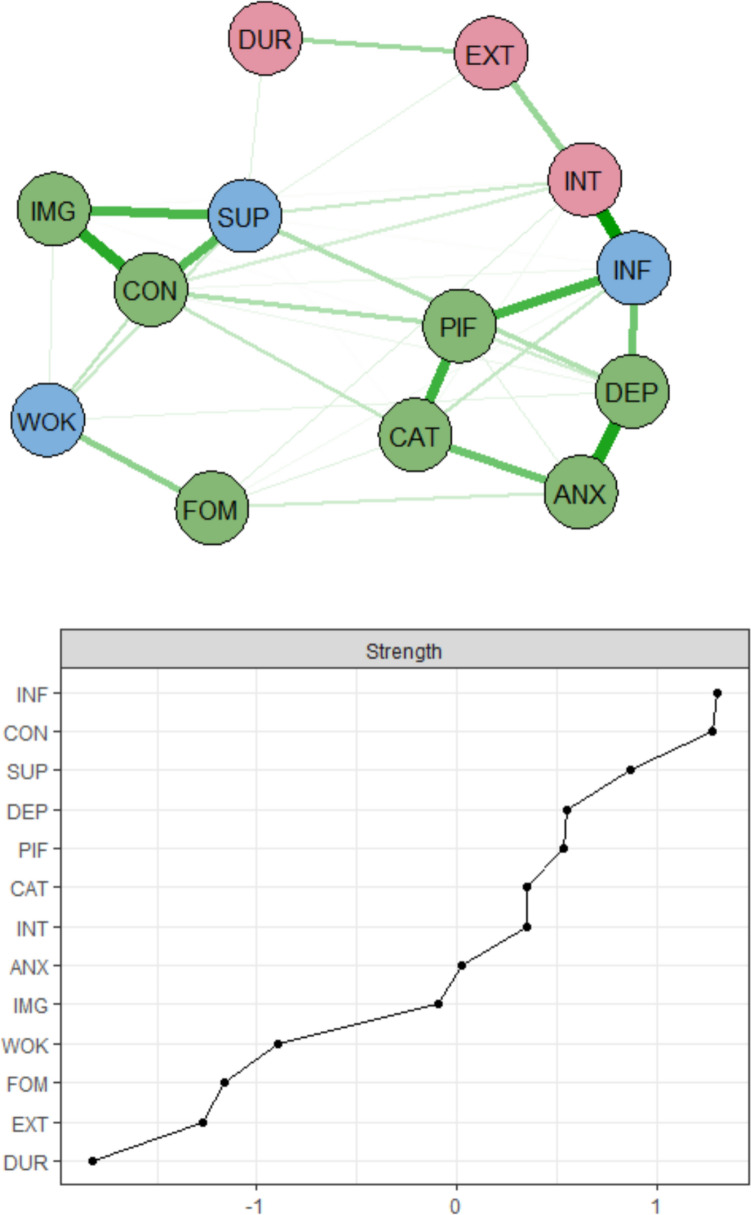
Network with strength centrality plot. There are three domains in nodes: red = biological nodes, green = psychological nodes, blue = social nodes. In the strength centrality plot, *z*-scores are shown on the *x*-axis rather than raw centrality indices. *INT* pain intensity (NRS), *EXT* number of pain locations, *DUR* pain duration, *INF* pain interference (MPI), *DEP* depression (HADS), *ANX* anxiety (HADS), *WOK* work life (EHP-30), *SUP* social support (EHP-30), *CON* control and powerlessness (EHP-30), *IMG* self-image (EHP-30), *PIF* psychological inflexibility (PIPS), *CAT* pain catastrophizing (PCS), *FOM* fear of movement (TAMPA)

When the psychological process nodes were added (Fig. 3), the EHP-30-based cluster remained, but pain catastrophizing and psychological inflexibility were incorporated into the first cluster, together with pain aspects and negative emotions.

Control and powerlessness and social support remained the leading nodes, but pain interference ranked higher than both. Interestingly, psychological inflexibility, pain catastrophizing, and fear of movement showed different connectivities with other nodes. Fear of movement was positioned closer to the work life subscale of the EHP-30, whereas psychological inflexibility and pain catastrophizing clustered more closely with depression, anxiety, and pain interference. Among the psychological process variables, psychological inflexibility exhibited comparatively higher strength centrality than the others. Fear of movement emerged as the least important psychological process node in the network. Visually, psychological inflexibility showed a strong and direct connection with pain interference, while pain catastrophizing was strongly connected with anxiety.

## Discussion

There is a need for a multidimensional understanding of endometriosis and for more targeted, personalized treatment interventions, including psychological approaches. The first aim of this study was to identify key biopsychosocial characteristics in individuals attending a tertiary clinic for endometriosis. One major finding was that psychosocial variables emerged as the most central elements in this biopsychosocial symptom network, when the psychological process variables were not included. The strongest variable was control and powerlessness, closely followed by social support and depression. These factors appear to be central targets for intervention in endometriosis, suggesting that enhancing perceived control, strengthening social support, and alleviating depressive symptoms may promote better overall adjustment.

These findings are consistent with previous research showing that depression is a common comorbidity in endometriosis [8, 63, 64] and that social support serves as an important resilience factor [65]. Both social support and perceived control have been associated with quality of life in individuals with endometriosis, and improvements in these factors have been observed following psychological treatment [66]. Because such processes are often targeted in psychological therapies, such as CBT [28], these results suggest that psychological interventions may be of particular value in endometriosis. This is consistent with recent meta-analytic evidence emphasizing the potential of psychological treatments to reduce pain and improve mental and physical well-being in this population [27]. However, current international guidelines (e.g., [15]) conclude that, at present, no firm recommendations can be made regarding the effectiveness of psychological interventions for improving pain or quality of life in individuals with endometriosis, highlighting the need for further rigorous research in this area [15].

In contrast, biological factors such as pain extent and pain duration showed lower centrality in the biopsychosocial network, suggesting they may play a less integrative role in the symptom structure compared to psychosocial factors. This pattern could indicate that once pain becomes chronic, its established features are less intertwined with pain-related functioning as compared to psychological processes, such as perceived control, catastrophizing, or depression, which exert broader influences across the network. These findings contrast with current treatment approaches for endometriosis, which are typically biomedical [12, 13], but are in line with previous studies in other chronic pain populations, where pain extent has shown limited relevance when outlining symptom structures and understanding functioning [42, 67].

The second aim of this study was to inform the development of future, targeted, and efficacious psychological interventions by examining the importance of psychological processes central to two scientific models, pain catastrophizing and fear of movement (the fear-avoidance model), and psychological inflexibility (the psychological flexibility model). Psychological inflexibility emerged as the most central psychological process in the network, ranking among the stronger nodes. These findings are in accordance with the scarce existing research on endometriosis, where a single study has demonstrated that psychological flexibility, and its counterpart psychological inflexibility, are relevant to functioning in individuals with endometriosis [26]. These findings align with previous studies, where therapeutic processes from the psychological flexibility model have been identified as essential for functioning in chronic pain populations [47, 68–70] and have been shown to be more influential than fear of movement for treatment outcomes in chronic pain interventions [71]. The psychological flexibility model emphasizes healthy activity and well-being achieved through increases in psychological flexibility and Acceptance and Commitment Therapy (ACT) is based on this model [72]. Psychological flexibility involves the ability to act in alignment with one’s values despite psychological or physical discomfort, whereas psychological inflexibility represents difficulty engaging in meaningful activities due to avoidance or fusion with distressing internal experiences. Psychological flexibility entails six core therapeutic processes: acceptance, cognitive defusion, present-focused awareness, self-as-context, committed action, and value-based action, which can be summarized as behavioral patterns that are open, aware, and engaged [73]. Research on the psychological flexibility model and ACT has grown substantially in recent years, and empirical support for this model and treatment approach in chronic pain is robust [68]. Moreover, a recent study suggests that such treatment strategies may also be beneficial for individuals with endometriosis [66]. Overall, the study enhances our knowledge of the psychological factors involved in endometriosis and provides a foundation for developing and testing targeted psychological interventions. Based on the findings of this study, interventions focused on psychological inflexibility appear particularly promising for individuals with endometriosis, as they may improve functioning, reduce avoidance behaviors, and enhance engagement in valued activities. Building on these findings, future studies could empirically test this approach through well-designed pilot studies or randomized controlled trials, assessing changes in pain, physical functioning, quality of life, and psychological flexibility. Furthermore, investigating which subgroups, such as patients with varying symptom severity, care settings, or diagnostic status, benefit most could help guide tailored intervention strategies.

Even if the relevance of pain catastrophizing was not as strong as psychological inflexibility, it still appears to be a meaningful treatment target in endometriosis. Catastrophizing is a core component from the fear-avoidance model, reflecting a tendency to amplify negative thoughts and emotions related to pain [52, 74]. In chronic pain populations, such as those with fibromyalgia, pain catastrophizing has been associated with greater pain intensity, emotional distress, disability, and poorer treatment outcomes [75–77]. The significance of pain catastrophizing in endometriosis has also been highlighted in earlier studies [34, 35], underscoring its role as a key factor in pain-related functioning [28, 78]. Fear of movement showed low relevance in this symptom network, which contrasts with previous research emphasizing its importance in chronic pain populations [29, 79]. One possible explanation for this discrepancy is that endometriosis-related pain may be less directly linked to movement or physical activity compared to musculoskeletal pain conditions, where movement often triggers pain and reinforces avoidance behaviors.

Strengths of the study included the selection of the population, as only patients with laparoscopically or ultrasound-verified endometriosis were included, and the inclusion of a wide array of aspects from the biopsychosocial model in the analyses. Nonetheless, the present findings must be interpreted in light of certain limitations. All measures were self-reported, which may introduce response biases or common method variance that could affect the observed associations between variables and the interpretation of centrality findings. Moreover, all measures included were organized according to the biopsychosocial model, with most factors being psychological, which may have influenced the results. The cross-sectional design of the study precluded the identification of causal directions. The study sample was relatively small and drawn from a tertiary care clinical service, with some participants scheduled for surgical interventions. Consequently, the results may not fully reflect symptom patterns in patients treated at primary or secondary care settings, or in individuals without a formal diagnosis of endometriosis, who may differ in symptom severity, treatment history, or other clinical characteristics. The self-report measure PIPS was used to measure psychological inflexibility. While the PIPS has satisfactory psychometric properties [53], cross-validation studies suggest that the total score may not fully capture all aspects of psychological flexibility/inflexibility, such as present-focused awareness, self-as-context, committed action, and value-based action [80, 81]. Future research could address these limitations by employing alternative or complementary measures of psychological flexibility and recruiting larger, more diverse samples from different levels of care to enhance the representativeness and generalizability of the findings.

In conclusion, a biopsychosocial approach appears to be particularly useful in understanding endometriosis. Despite the current treatment regimen being predominately biomedical, psychosocial variables, such as perceived control and powerlessness, social support, and depression were especially important for the symptom structure in this patient population with laparoscopically or ultrasound-verified endometriosis. When aiming to inform future, promising psychological interventions for endometriosis, psychological inflexibility emerged as the most central psychological process in the network. Interventions based on this model, such as ACT, may hold promise for improving outcomes in this population, but this requires testing in future studies.

## Data Availability

Due to ethical restrictions the research data supporting the results of the manuscript cannot be shared.

## References

[1] Zondervan KT, Becker CM, Missmer SA (2020) Endometriosis. N Engl J Med 382(13):1244–1256 32212520 10.1056/NEJMra1810764

[2] Vercellini P et al (2014) Endometriosis: pathogenesis and treatment. Nat Rev Endocrinol 10(5):261–275 24366116 10.1038/nrendo.2013.255

[3] As-Sanie S et al (2025) Endometriosis: a review. JAMA 334(1):64–78 40323608 10.1001/jama.2025.2975 PMC13498397

[4] Stratton P et al (2015) Association of chronic pelvic pain and endometriosis with signs of sensitization and myofascial pain. Obstet Gynecol 125(3):719–728 25730237 10.1097/AOG.0000000000000663 PMC4347996

[5] Culley L et al (2013) The social and psychological impact of endometriosis on women’s lives: a critical narrative review. Hum Reprod Update 19(6):625–639 23884896 10.1093/humupd/dmt027

[6] Della Corte L et al (2020) The burden of endometriosis on women’s lifespan: a narrative overview on quality of life and psychosocial wellbeing. Int J Environ Res Public Health. 10.3390/ijerph17134683 32610665 10.3390/ijerph17134683 PMC7370081

[7] Nnoaham KE et al (2011) Impact of endometriosis on quality of life and work productivity: a multicenter study across ten countries. Fertil Steril 96(2):366-373.e8 21718982 10.1016/j.fertnstert.2011.05.090 PMC3679489

[8] Gao M et al (2020) Psychiatric comorbidity among women with endometriosis: nationwide cohort study in Sweden. Am J Obstet Gynecol 223(3):415.e1-415.e16 32112731 10.1016/j.ajog.2020.02.033

[9] Delanerolle G et al (2021) A systematic review and meta-analysis of the Endometriosis and Mental-Health Sequelae; The ELEMI Project. Womens Health 17:17455065211019717 10.1177/17455065211019717 PMC8182632 34053382

[10] Gatchel RJ et al (2007) The biopsychosocial approach to chronic pain: scientific advances and future directions. Psychol Bull 133(4):581–624 17592957 10.1037/0033-2909.133.4.581

[11] Sarria-Santamera A et al (2023) A novel classification of endometriosis based on clusters of comorbidities. Biomedicines. 10.3390/biomedicines11092448 37760889 10.3390/biomedicines11092448 PMC10525703

[12] Vercellini P et al (2009) Endometriosis: current therapies and new pharmacological developments. Drugs 69(6):649–675 19405548 10.2165/00003495-200969060-00002

[13] National Institute for Health and Care Excellence (2017) Endometriosis: diagnosis and management (NG73). NICE, London; 2022. https://www.nice.org.uk/guidance/ng73 29787038

[14] Giudice LC, Kao LC (2004) Endometriosis. Lancet 364(9447):1789–1799 15541453 10.1016/S0140-6736(04)17403-5

[15] Becker CM et al (2022) ESHRE guideline: endometriosis. Hum Reprod Open 2022(2):hoac009 35350465 10.1093/hropen/hoac009 PMC8951218

[16] Turk DC (2002) Clinical effectiveness and cost-effectiveness of treatments for patients with chronic pain. Clin J Pain 18(6):355–365 12441829 10.1097/00002508-200211000-00003

[17] McCracken LM, Turk DC (2002) Behavioral and cognitive-behavioral treatment for chronic pain—outcome, predictors of outcome, and treatment process. Spine 27:2564–2573 12435995 10.1097/00007632-200211150-00033

[18] Hornberger J et al (2008) Rechargeable spinal cord stimulation versus non-rechargeable system for patients with failed back surgery syndrome: a cost-consequences analysis. Clin J Pain 24(3):244–252 18287831 10.1097/AJP.0b013e318160216a

[19] Taylor RS (2006) Spinal cord stimulation in complex regional pain syndrome and refractory neuropathic back and leg pain/failed back surgery syndrome: results of a systematic review and meta-analysis. J Pain Symptom Manage 31(4 Suppl):S13–S19 16647590 10.1016/j.jpainsymman.2005.12.010

[20] Turk DC, Wilson HD, Cahana A (2011) Treatment of chronic non-cancer pain. Lancet 377(9784):2226–2235 21704872 10.1016/S0140-6736(11)60402-9

[21] Grundström H (2018) Disclosing the invisible-experiences, outcomes and quality of endometriosis healthcare. Department of Medical and Health Sciences, Linköping University, Sweden

[22] Colvin LA, Fallon MT (2010) Opioid-induced hyperalgesia: a clinical challenge. Br J Anaesth 104(2):125–127 20086062 10.1093/bja/aep392

[23] Edlund MJ et al (2014) The role of opioid prescription in incident opioid abuse and dependence among individuals with chronic noncancer pain: the role of opioid prescription. Clin J Pain 30(7):557–564 24281273 10.1097/AJP.0000000000000021 PMC4032801

[24] van Stein K et al (2023) Understanding psychological symptoms of endometriosis from a research domain criteria perspective. J Clin Med. 10.3390/jcm12124056 37373749 10.3390/jcm12124056 PMC10299570

[25] Williams ACC et al (2020) Psychological therapies for the management of chronic pain (excluding headache) in adults. Cochrane Database Syst Rev 8(8):Cd007407 32794606 10.1002/14651858.CD007407.pub4 PMC7437545

[26] Sundström FTA et al (2023) Associations between psychological flexibility and daily functioning in endometriosis-related pain. Scand J Pain. 10.1515/sjpain-2022-0157 37867345 10.1515/sjpain-2022-0157

[27] Del Pino-Sedeño T et al (2024) Effectiveness of psychological interventions in endometriosis: a systematic review with meta-analysis. Front Psychol 15:1457842 39529727 10.3389/fpsyg.2024.1457842 PMC11551779

[28] McCracken LM, Morley S (2014) The psychological flexibility model: a basis for integration and progress in psychological approaches to chronic pain management. J Pain 15(3):221–234 24581630 10.1016/j.jpain.2013.10.014

[29] Vlaeyen J, Linton S (2000) Fear-avoidance and its consequences in musculoskeletal pain: a state of the art. Pain 85:317–332 10781906 10.1016/S0304-3959(99)00242-0

[30] Vlaeyen JWS et al (2001) Graded exposure in vivo in the treatment of pain-related fear: a replicated single-case experimental design in four patients with chronic low back pain. Behav Res Ther 39(2):151–166 11153970 10.1016/s0005-7967(99)00174-6

[31] Russek L et al (2015) A cross-sectional survey assessing sources of movement-related fear among people with fibromyalgia syndrome. Clin Rheumatol 34(6):1109–1119 24481649 10.1007/s10067-014-2494-5

[32] Linton SJ (2013) A transdiagnostic approach to pain and emotion. J Appl Biobehav Res 18(2):82–103 24143062 10.1111/jabr.12007 PMC3796860

[33] Hughes LS et al (2017) Acceptance and commitment therapy (ACT) for chronic pain: a systematic review and meta-analyses. Clin J Pain 33(6):552–568 27479642 10.1097/AJP.0000000000000425

[34] Martin CE et al (2011) Catastrophizing: a predictor of persistent pain among women with endometriosis at 1 year. Hum Reprod 26(11):3078–3084 21900393 10.1093/humrep/der292 PMC3196877

[35] McPeak AE et al (2018) Pain catastrophizing and pain health-related quality-of-life in endometriosis. Clin J Pain 34(4):349–356 28731958 10.1097/AJP.0000000000000539

[36] Åkerblom S (2018) Predictors and mediators of outcome in CBT for chronic pain: the roles of psychological flexibility and PTSD. Department of Psychology, Lund University, Lund

[37] Escriva-Boulley G et al (2023) Effects of a physical activity and endometriosis-based education program delivered by videoconference on endometriosis symptoms: the CRESCENDO program (inCRease physical Exercise and Sport to Combat ENDOmetriosis) protocol study. Trials 24(1):759 38012776 10.1186/s13063-023-07792-1 PMC10680283

[38] Borsboom D (2017) A network theory of mental disorders. World Psychiatry 16(1):5–13 28127906 10.1002/wps.20375 PMC5269502

[39] Cervin M et al (2019) The centrality of doubting and checking in the network structure of obsessive-compulsive symptom dimensions in youth. J Am Acad Child Adolesc Psychiatry 59(7):880–889 31421234 10.1016/j.jaac.2019.06.018 PMC7219532

[40] Fried EI et al (2018) Replicability and generalizability of posttraumatic stress disorder (PTSD) networks: a cross-cultural multisite study of PTSD symptoms in four trauma patient samples. Clin Psychol Sci 6(3):335–351 29881651 10.1177/2167702617745092 PMC5974702

[41] Cramer AO et al (2016) Major depression as a complex dynamic system. PLoS ONE 11(12):e0167490 27930698 10.1371/journal.pone.0167490 PMC5145163

[42] Åkerblom S et al (2021) A network analysis of clinical variables in chronic pain: a study from the Swedish Quality Registry for Pain Rehabilitation (SQRP). Pain Med 22(7):1591–1602 33706371 10.1093/pm/pnaa473

[43] Ferreira-Valente MA, Pais-Ribeiro JL, Jensen MP (2011) Validity of four pain intensity rating scales. Pain 152(10):2399–2404 21856077 10.1016/j.pain.2011.07.005

[44] Jensen MP, Karoly P (1992) Self-report scales and procedures for assessing pain in adults. In: Turk DC, Melzack R (eds) Handbook of pain assessment. The Guildford Press, New York, London

[45] Rudy TE et al (1989) Multiaxial assessment of pain: multidimensional pain inventory. Computer program user’s manual. Version 2.1. Pain Evaluation and Treatment Institute, Pittburgh, PA

[46] Kerns RD, Rudy TE, Turk DC (1985) The West Haven-Yale Multidimensional Pain Inventory (WHYMPI). Pain 23(4):345–356 4088697 10.1016/0304-3959(85)90004-1

[47] Åkerblom S et al (2015) The mediating role of acceptance in multidisciplinary cognitive-behavioral therapy for chronic pain. J Pain 16(7):606–615 25840330 10.1016/j.jpain.2015.03.007

[48] Jones G et al (2001) Development of an endometriosis quality-of-life instrument: the Endometriosis Health Profile-30. Obstet Gynecol 98(2):258–264 11506842 10.1016/s0029-7844(01)01433-8

[49] Grundström H et al (2020) Psychometric evaluation of the Swedish version of the 30-item endometriosis health profile (EHP-30). BMC Womens Health 20(1):204 32928218 10.1186/s12905-020-01067-6 PMC7490900

[50] Zigmond AS, Snaith RP (1983) The hospital anxiety and depression scale. Acta Psychiatr Scand 67(6):361–370 6880820 10.1111/j.1600-0447.1983.tb09716.x

[51] Lisspers J, Nygren A, Soderman E (1997) Hospital anxiety and depression scale (HAD): some psychometric data for a Swedish sample. Acta Psychiatr Scand 96:281–286 9350957 10.1111/j.1600-0447.1997.tb10164.x

[52] Sullivan MJL, Bishop SR, Pivik J (1995) The pain catastrophizing scale: development and validation. Psychol Assess 7(4):524–532

[53] Wicksell RK et al (2010) The Psychological Inflexibility in Pain Scale (PIPS) - statistical properties and model fit of an instrument to assess change processes in pain related disability. Eur J Pain 14(7):771.e1-771.e14 20106685 10.1016/j.ejpain.2009.11.015

[54] Vlaeyen JWS et al (1995) Fear of movement/(re)injury in chronic low back pain and its relation to behavioral performance. Pain 62:363–372 8657437 10.1016/0304-3959(94)00279-N

[55] Swinkels-Meewisse IEJ et al (2003) Fear of movement/(re)injury, disability and participation in acute low back pain. Pain 105:371–379 14499456 10.1016/s0304-3959(03)00255-0

[56] Roelofs J et al (2007) Fear of movement and (re)injury in chronic musculoskeletal pain: evidence for an invariant two-factor model of the Tampa Scale for Kinesiophobia across pain diagnoses and Dutch, Swedish, and Canadian samples. Pain 131(1–2):181–190 17317011 10.1016/j.pain.2007.01.008

[57] Borsboom D (2008) Psychometric perspectives on diagnostic systems. J Clin Psychol 64(9):1089–1108 18683856 10.1002/jclp.20503

[58] Friedman J, Hastie T, Tibshirani R (2008) Sparse inverse covariance estimation with the graphical lasso. Biostatistics 9(3):432–441 18079126 10.1093/biostatistics/kxm045 PMC3019769

[59] Liu H, Lafferty J, Wasserman L (2009) The nonparanormal: semiparametric estimation of high dimensional undirected graphs. J Mach Learn Res 10:2295–2328 PMC4729207 26834510

[60] Bringmann LF et al (2019) What do centrality measures measure in psychological networks? J Abnorm Psychol 128(8):892–903. 10.1037/abn0000446 31318245 10.1037/abn0000446

[61] Epskamp S et al (2012) Qgraph: network visualizations of relationships in psychometric data. J Stat Softw 48(4):1–18

[62] Epskamp S, Borsboom D, Fried EI (2018) Estimating psychological networks and their accuracy: a tutorial paper. Behav Res Methods 50(1):195–212 28342071 10.3758/s13428-017-0862-1 PMC5809547

[63] Laganà AS et al (2017) Anxiety and depression in patients with endometriosis: impact and management challenges. Int J Womens Health 9:323–330 28553145 10.2147/IJWH.S119729 PMC5440042

[64] Pope CJ et al (2015) A systematic review of the association between psychiatric disturbances and endometriosis. J Obstet Gynaecol Can 37(11):1006–1015 26629721 10.1016/s1701-2163(16)30050-0

[65] Schwab R et al (2022) Mental health and social support are key predictors of resilience in German women with endometriosis during the COVID-19 pandemic. J Clin Med. 10.3390/jcm11133684 35806968 10.3390/jcm11133684 PMC9267240

[66] Hansen KE et al (2023) Psychological interventions improve quality of life despite persistent pain in endometriosis: results of a 3-armed randomized controlled trial. Qual Life Res 32(6):1727–1744 36797461 10.1007/s11136-023-03346-9 PMC10172241

[67] Gerdle B et al (2019) Who benefits from multimodal rehabilitation—an exploration of pain, psychological distress, and life impacts in over 35,000 chronic pain patients identified in the Swedish Quality Registry for Pain Rehabilitation. J Pain Res 12:891–908 30881099 10.2147/JPR.S190003 PMC6411315

[68] McCracken LM (2024) Psychological flexibility, chronic pain, and health. Annu Rev Psychol 75:601–624 37585667 10.1146/annurev-psych-020223-124335

[69] Åkerblom S et al (2019) Acceptance: a factor to consider in persistent pain after neck trauma. Scand J Pain. 10.1515/sjpain-2019-0021 31203263 10.1515/sjpain-2019-0021

[70] Åkerblom S et al (2021) Predictors and mediators of outcome in cognitive behavioral therapy for chronic pain: the contributions of psychological flexibility. J Behav Med 44(1):111–122 32642875 10.1007/s10865-020-00168-9 PMC7846536

[71] Gerdle B et al (2024) Acceptance and fear-avoidance mediate outcomes of interdisciplinary pain rehabilitation programs at 12-month follow-up: a clinical registry-based longitudinal cohort study from the Swedish Quality Registry for Pain Rehabilitation (SQRP). J Pain Res 17:83–105 38196970 10.2147/JPR.S438260 PMC10775695

[72] Hayes SC, Strosahl KD, Wilson KG (1999) Acceptance and commitment therapy : an experiential approach to behavior change. Guilford Press, New York, London

[73] Hayes SC, Vilatte M, Levin M, Hildebrandt M (2011) Open, aware, and active: contextual approaches as an emerging trend in the behavioral and cognitive therapies. Annu Rev Clin Psychol 7:141–168 21219193 10.1146/annurev-clinpsy-032210-104449

[74] Vlaeyen JW, Linton SJ (2000) Fear-avoidance and its consequences in chronic musculoskeletal pain: a state of the art. Pain 85(3):317–332 10781906 10.1016/S0304-3959(99)00242-0

[75] Edwards RR et al (2016) The role of psychosocial processes in the development and maintenance of chronic pain. J Pain 17(9 Suppl):T70-92 27586832 10.1016/j.jpain.2016.01.001 PMC5012303

[76] Martinez-Calderon J et al (2019) Pain catastrophizing and function in individuals with chronic musculoskeletal pain: a systematic review and meta-analysis. Clin J Pain 35(3):279–293 30664551 10.1097/AJP.0000000000000676

[77] Shimada S et al (2025) A systematic review of pain catastrophizing and chronic musculoskeletal pain. Pain Manage Nurs. 10.1016/j.pmn.2025.07.014 10.1016/j.pmn.2025.07.014 PMC13447084 40858413

[78] Petrini L, Arendt-Nielsen L (2020) Understanding pain catastrophizing: putting pieces together. Front Psychol 11:603420 33391121 10.3389/fpsyg.2020.603420 PMC7772183

[79] Leeuw M et al (2007) The fear-avoidance model of musculoskeletal pain: current state of scientific evidence. J Behav Med 30(1):77–94 17180640 10.1007/s10865-006-9085-0

[80] Trompetter HR et al (2014) The Psychological Inflexibility in Pain Scale (PIPS): exploration of psychometric properties in a heterogeneous chronic pain sample. Eur J Psychol Assess 30(4):289–295

[81] Barke A et al (2015) The psychological inflexibility in pain scale (PIPS)—validation, factor structure and comparison to the chronic pain acceptance questionnaire (CPAQ) and other validated measures in German chronic back pain patients. BMC Musculoskelet Disord 16(1):1–10 26215038 10.1186/s12891-015-0641-z PMC4517641

